# Parental psychological distress and child maladjustment: Exploring the moderating role of sibling relationship quality

**DOI:** 10.3389/fpsyg.2022.968985

**Published:** 2022-08-16

**Authors:** Jessica Turgeon, Jean-François Bureau

**Affiliations:** ^1^Department of Psychology, Université du Québec à Trois-Rivières, Trois-Rivières, QC, Canada; ^2^School of Psychology, University of Ottawa, Ottawa, ON, Canada

**Keywords:** paternal psychological distress, maternal psychological distress, child social-emotional maladjustment, siblings, sibling relationship quality

## Abstract

The aim of this study was to investigate whether the quality of the sibling relationship moderates the association between parental psychological distress and child maladjustment (i.e., internalizing and externalizing problems). We extended previous literature by studying mothers and fathers separately and by including an observational measure of the quality of the sibling relationship. Participants were 52 two-parent families from a community sample who had at least two children living at home. Only one child (aged 6–10 years) was targeted for the study and studied in relation to his/her siblings. Mothers and fathers completed a self-reported questionnaire on their psychological distress and individually assessed their child’s social-emotional maladjustment. The targeted child’s interactions with his/her siblings were observed by independent judges during a home-visit. Results indicate that both maternal and paternal psychological distress are significant predictors of child social-emotional maladjustment. Moderation analyses reveal that children of distressed fathers are at lower risk of social-emotional maladjustment when they engage in highly positive interactions with their siblings. *Post hoc* analyses suggest that only sibling empathy (not teaching nor companionship) is a significant moderator of the association between paternal psychological distress and child maladjustment. The results of this study provide further evidence of the influence that fathers have in their child’s development and highlight the importance of using a systemic family approach to promote children’s social-emotional adaptation in the context of parental distress.

## Introduction

There is considerable evidence that parental psychological distress is a significant predictor of child social-emotional maladjustment, as evidenced by internalizing (e.g., depression, anxiety) and externalizing (e.g., hyperactivity, aggression) problems (Connell and Goodman, 2002). The small magnitude of effect sizes for mothers and fathers found in Connell and Goodman’s (2002) meta-analytic review, however, indicates that parental mental health problems only account for a small proportion of variance in children’s internalizing and externalizing behavior problems. These results suggest that there are likely moderating variables at play, which may increase or decrease the negative effects of parental psychological distress on child maladjustment. To date, most studies have focussed on parenting behaviors (e.g., harsh physical or psychological behaviors toward the child) or the quality of parent-child interactions (e.g., reciprocal or conflictual interactions) as potential transmission mechanisms (e.g., Dubois-Comtois et al., 2013; Villodas et al., 2018). While it is well-recognized that the quality of the parent-child relationship influences child development, the *Family Systems Theory* highlights the importance of viewing the individual as part of a complex and dynamic system composed of multiple subsystems that each share unique characteristics (Minuchin, 1974, 1985; Cox and Paley, 2003; Volling, 2005). One important subsystem that has often been neglected in the literature is the sibling relationship. Nonetheless, the results of a literature review indicate that siblings can influence each other’s development and adjustment (McHale et al., 2012). In accordance with this theoretical framework, Keeton et al. (2015) examined the quality of the sibling relationship as a potential moderator of the association between parental psychological distress (62 mothers, 19 fathers) and child psychological adjustment. Results suggest that children with more positive sibling relationships are at lower risk of developing social-emotional problems in the context of parental distress than children with lower quality sibling relationships (Keeton et al., 2015). One of the limitations of this study, however, is that fathers were not distinguished from mothers in the analyses. Consequently, it is not possible to determine whether the results obtained apply equally to fathers and mothers or whether the effect is primarily driven by mothers who account for 76.5% of the sample. The current study aims to extend these findings by conducting separate analyses for mothers and fathers and by including an observational measure of the sibling relationship. These findings will likely contribute to our understanding of how positive sibling relationships may protect children against the risks associated with maternal and paternal psychological distress.

### Parental psychological distress and child maladjustment

Most studies investigating the association between parental psychological distress and child maladjustment have measured parental distress in terms of depression and/or anxiety symptoms, psychopathology, or parenting stress which, in turn, have been associated with interpersonal difficulties (Girard et al., 2017). A meta-analysis by Connell and Goodman (2002) reviewed 134 studies combining clinical and community samples with children under 18 years old (*M*_age_ = 9 years). Results revealed that maternal and paternal psychological distress were related to child internalizing and externalizing behavior problems (Connell and Goodman, 2002). Whereas maternal psychopathology was a stronger predictor of child emotional and behavioral problems in younger children (≤6 years), paternal psychopathology was more predictive of child emotional and behavioral problems in older children (≥13 years). Another meta-analysis by Goodman et al. (2011) combining data from 193 studies found that maternal depression was associated with more negative child outcomes (i.e., internalizing and externalizing behaviors, general psychopathology, negative affect and behavior) and less positive child outcomes (i.e., positive affect and behavior). Of importance, this review excluded studies conducted with fathers as well as those combining data from mothers and fathers. Other studies have found that mothers’ self-reported symptoms of depression are associated with lower school achievement and physical aggression in children between the ages of 4 and 5, anxiety symptoms in children between the ages of 10 and 11 (Letourneau et al., 2013), and an increased risk for depression at ages 16 and 18 (Murray et al., 2011; Pearson et al., 2013). In Bureau et al.’s (2009) longitudinal study, maternal depressive symptoms measured in infancy were a significant predictor of depressive symptoms in children at ages 8 and 19 years, even after controlling for chronic or concurrent maternal depressive symptoms in middle childhood.

In terms of anxiety, a recent meta-analysis of 25 studies (Lawrence et al., 2019) revealed that offspring of parents with an anxiety disorder are almost twice more likely (risk ratio = 1.76) to develop an anxiety disorder, and almost one and a half times (risk ratio = 1.31) more likely to develop a depressive disorder compared to offspring of parents without such a diagnosis, which is consistent with findings from a prior meta-analysis (Micco et al., 2009). Both these reviews combined data from mothers and fathers indiscriminately, thus limiting our understanding of their individual effects. Nonetheless, these findings, overall, suggest a positive link between parental psychological distress, measured by indicators of depression and/or anxiety, and child emotional and behavioral problems. Similar results have also been found in relation to different measures of parental psychological distress, such as parenting stress and maternal distress (Barry et al., 2005), maternal cumulative stress (Crnic et al., 2005), mothers’ and fathers’ symptoms of depression, anxiety, and hostility (Papp et al., 2005), and parents’ symptoms of depression, anxiety, loneliness, and lack of resilience (Sun et al., 2022).

### Paternal psychological distress and child maladjustment

Although most studies have been conducted solely with mothers or with both parents indiscriminately, results from studies in which separate analyses were performed for mothers and fathers suggest that paternal distress is also a significant predictor of child maladjustment. For instance, Low and Stocker’s (2005) path analyses results revealed a direct link between fathers’ depressive symptoms and children’s externalizing behavior problems at age 10, whereas no direct association was found between mothers’ depressive symptoms and child behavior problems. More recently, Vallotton et al. (2016) examined child behavior problems in relation to both parents’ depressive symptoms and parenting stress. Results from this study, conducted with low-income families, found that child behavior problems at age 3 were significantly related to mothers’ and father’s ratings of depression and parenting stress. By fifth grade, both parents’ depressive symptoms were significantly related to higher levels of child hyperactivity, whereas only fathers’ depressive symptoms were related to lower levels of child cooperation (Vallotton et al., 2016). In a recent study, Dubois-Comtois et al. (2021) investigated the link between fathers’ self-reported psychological distress and child behavior problems assessed by both parents. Results revealed that higher levels of paternal psychological distress were related to higher levels of internalizing and externalizing behavior problems in young children (3–5 years old) from low-SES families. Together, these results provide evidence of a positive association between both maternal and paternal psychological distress and child maladjustment. Researchers have explored this relation further with the aim of identifying transmission mechanisms as well as factors that may protect children against the effects of parental distress.

### Explanatory mechanisms linking parental distress and child adjustment

A seminal review article by Goodman and Gotlib (1999) suggests that both genetic and environmental factors are involved in the association between parental psychological distress and children’s social-emotional adjustment. Researchers have placed a particular emphasis on the study of environmental factors, as they can be targeted in prevention and intervention programs. One contextual factor that has received much attention is the parent-child relationship, namely the quality of the relationship itself or the way in which parents behave toward their child. A recent meta-analysis investigating the mediating effect of parenting behaviors on the relation between maternal depression and adverse child functioning found that more negative behaviors and less positive behaviors help explain this association (Goodman et al., 2020). In Dubois-Comtois et al.’s (2021) study, father’s psychological distress was associated with less positive behaviors (i.e., availability and affection) and more negative behaviors (i.e., negativity, irritation, confrontation) during a parent-child play interaction. The relation between paternal distress and child behavior problems was partially mediated by the quality of father-child interactions.

Altogether, these studies show that distressed parents have more difficulty engaging in positive behaviors toward their child which, in turn, influences the child’s social-emotional adjustment. What remains unclear, however, is whether individual or environmental factors have the potential to protect children in the context of parental psychological distress. Although it receives little empirical attention, the sibling relationship is one factor that could potentially influence the transmission of psychological symptoms from parents to their offspring. One may wonder: are children of distressed parents at lower risk of maladaptation when they have supportive and empathetic relationships with their siblings on whom they can rely during difficult times? The *Family Systems Theory* highlights the importance of viewing families as being composed of both autonomous and interdependent subsystems, in which siblings play a unique role in shaping the child’s development (Minuchin, 1974; Cox and Paley, 2003). Existing literature suggests that siblings continuously influence one another and can provide a source of emotional safety for the child and a foundation for emotional regulation (Whiteman et al., 2011). Additional research is needed to understand how siblings affect child adjustment in the context of parental risk.

### Sibling relationships

Numerous studies show that siblings share a unique bond (Dunn, 2002) and play an important role in children’s social and emotional development (Dunn, 2014; Howe and Recchia, 2014; Kramer, 2014; Buist et al., 2019; Jambon et al., 2019). Whereas positive sibling relationships have been associated with positive child outcomes, negative sibling relationships have been linked to a variety of adaptative functioning difficulties both across childhood and adolescence. For instance, a longitudinal study found that sibling negativity is associated with higher levels of depressive symptoms and more behavior problems in adolescence (Whiteman et al., 2015). Moreover, a study examining the influence of sibling relationship quality on child outcomes in middle childhood found that children with conflictual relationships self-reported more internalizing and externalizing behaviors compared to children with harmonious sibling relationships (Buist and Vermande, 2014). Based on meta-analytic results, sibling relationships characterized by more warmth, less conflict and less differential treatment are associated with lower levels of internalizing and externalizing problems in childhood and adolescence (Buist et al., 2013). Finally, findings from a recent study indicate that more sibling conflict is related to higher levels of child externalizing problems (not internalizing problems) and lower levels of self-esteem 1 year later (van Dijk et al., 2022).

In light of these findings, researchers have explored whether the quality of the sibling relationship can act as a protective factor against child maladjustment. Findings from a variety of studies appear to support this hypothesis. Among the first studies exploring the protective role of siblings is a cross-sectional study by Jenkins and Smith (1990). Results from this study reveal that a good sibling relationship moderates the association between poor parental marriage and children’s emotional and behavioral problems. As sibling relationship quality increases, the strength of the association between conflictual parental marriage and child symptoms decreases (Jenkins and Smith, 1990). Research on the protective role of siblings has gained much attention in response to these findings, which continue to be supported by more recent studies using a variety of research designs. For instance, results from a longitudinal study by Gass et al. (2007) indicate that sibling affection plays a protective role in the relation between stressful life events reported by mothers and child internalizing symptoms, even after controlling for the quality of the mother-child relationship. Conversely, no moderation effect was found in relation to externalizing symptoms. More recently, researchers have found that having positive sibling relationships moderates the association between exposure to parental conflict and emotional insecurity (Davies et al., 2019) as well as between parental conflict and adolescents’ depressive symptoms (Tucker et al., 2013), both in expected directions. Higher levels of sibling support have also been found to buffer the negative effects of sibling conflict on children’s self-esteem following their parents’ divorce (van Dijk et al., 2022). In Keeton et al.’s (2015) study, the sibling relationship was investigated as a potential moderator of the association between parental psychological distress and child emotional and behavioral problems. Children were between the ages of 7 and 12 and had a parent with a clinical anxiety disorder (Keeton et al., 2015). Findings revealed that the association between parental reports of psychological distress and parental reports of child psychological problems was moderated by children’s ratings of low sibling companionship and high sibling conflict. Specifically, only children whose sibling relationships were characterized by lower levels of companionship and higher levels of conflicts had more emotional and behavioral problems as a consequence of parental psychological distress, suggesting that poor sibling relationships confer additional risk.

### The present study: Objectives and hypotheses

The present study was inspired by the findings of Keeton et al. (2015) and Dubois-Comtois et al. (2021) and aimed to investigate whether the quality of the sibling relationship plays a moderating role in the association between parental psychological distress and child social-emotional maladjustment. This study is the first to investigate this research question separately for mothers and fathers. While previous research has focused primarily on mothers, it is also important to include fathers, as they are increasingly involved with their children and are known to play an important role in their child’s social-emotional development (Cabrera et al., 2007, 2014; Pleck, 2010; Volling and Cabrera, 2019). Distressed fathers, however, may have more opportunity to withdraw than mothers as their roles are less socially prescribed (Cabrera et al., 2014), perhaps limiting the effects of distress on children. Another important contribution of this study is the inclusion of an observational measure of the quality of sibling interactions. While several previous studies have relied on parental self-reports, our study involved independent observers, as distressed parents may rate sibling interactions more negatively as a result of their symptoms.

The first objective of this study was to explore the influence of mothers’ and fathers’ psychological distress on child social-emotional maladjustment (i.e., internalizing and externalizing problems). Based on existing literature, we hypothesized that higher levels of maternal/paternal distress would be significantly related to higher levels of child maladjustment. The second objective of this study was to examine whether the quality of the sibling relationship moderates the association between maternal/paternal psychological distress and child maladjustment. First, without controlling for the effect of the other parent’s distress, and subsequently controlling for this effect. Based on Keeton et al.’s (2015) results, we hypothesized that parental psychological distress would be associated with higher levels of child maladjustment, and that this effect would be stronger in the context of more negative (i.e., rivalry, aggression, avoidance) and less positive (e.g., companionship, empathy, teaching) sibling interactions.

## Materials and methods

### Procedure

The present study was conducted as part of a longitudinal study with two data collection time points, separated by a 5-year gap. Families living in an Eastern city in Canada were recruited *via* radio, newspapers and online advertisements posted between 2009 and 2013. The original sample involved 157 heterosexual couples of children aged between 3 and 5 years old (intact families). After 5 years, families were contacted *via* phone or email and invited to participate in the second part of the longitudinal study. The child who participated at T1 remained the target child at T2. Although most families (*n* = 107; 68.15%) agreed to participate at time 2 (T2), only 63 families were composed of at least two children and agreed to participate in the sibling relationships assessment. Sibling interactions (i.e., the targeted child and all of his/her siblings) were observed during a home visit. Prior to this visit, both parents independently completed online questionnaires (i.e., a sociodemographic questionnaire, a parental distress questionnaire) and both parents completed a child outcome questionnaire. The analyses performed in this study are solely based on data collected at T2. The study was approved by the University’s Research Ethics Boards and a written informed consent was obtained from all participants.

#### Missing data

Of the 63 families who participated in the home visit for the sibling relationships assessment, 11 families did not complete all measures, resulting in 52 families with either complete or almost complete data. The percentage of missing data for these 52 families ranged from 2.23 to 4.45% for all variables under study (≤2 items missing per variable). As such, missing data were replaced using the *Expectation Maximization* method. Prior to replacing data, analyses were conducted for each measure to confirm that the missing data were missing completely at random.

### Participants

This study included 52 two-parent families with at least two children living at home. The sample consisted of heterosexual couples (47 married, 5 common-law) who had been together for approximately 15 years (SD = 3.7). All family members (parents, children, and the child’s siblings) were biologically related. Most families were not at socioeconomic risk. In fact, 73.1% (*n* = 38) reported having a gross annual family income of 100,000$ or more, although three families reported having a gross annual income below 39,999$. Fathers were between the ages of 29 and 55 years old (*M*_age_ = 41.02 years, *SD* = 5.3), whereas mothers were between the ages of 31 and 48 years old (*M*_age_ = 39.33 years, SD = 4.3). Additionally, 94.2% of fathers and 98.1% of mothers had a post-secondary education at the time of the data collection, with 28.8% of fathers and 30.8% of mothers having reached graduate studies. Most parents (84.6%) identified as White/Caucasian and were either English-speaking (69.2% fathers; 67.3% mothers) or French speaking (17.3% fathers; 21.2% mothers). As of children, only one child was targeted in each family. The targeted child had participated at T1 and was in middle childhood at T2 (*M*_age_ = 8.32 years, *Min* = 6.64; *Max* = 10.37; *SD* = 0.89; 33 girls). Among targeted children, 34 (65.4%) had only one sibling, whereas 12 had two siblings, five had three siblings and one child had four siblings. Of the 52 sets of siblings, 30 were mixed (male and female), 7 were composed of boys only and 15 were composed of girls only. Finally, among the 52 targeted children, 6 were the youngest of their siblings, 12 were the middle child, and 34 were the oldest of their siblings. The age gap between the youngest and oldest sibling ranged from less than a year (*n* = 12; 23.1%) to 6 years (*n* = 3; 5.8%).

### Measures

#### Sociodemographic questionnaire

At Time 2, mothers and fathers independently completed a sociodemographic questionnaire in which they were asked to answer questions about their marital status and family composition (e.g., number of children), their children (e.g., age, gender) as well as their socioeconomic situation (e.g., gross annual family income). Information about parents’ highest level of education, main spoken language, and ethnic background was also collected.

#### Parental psychological distress

Maternal and paternal psychological distress were assessed using a self-reported measure, the Outcome Questionnaire (OQ-45; Lambert et al., 2004a). The Outcome Questionnaire includes 45 items on a 5-point Likert scale, ranging from 0 (never) to 4 (almost always). It is composed of three subscales: (1) symptom distress (e.g., depression and anxiety), (2) problems in interpersonal relations (e.g., loneliness, interpersonal conflicts, marriage problems), and (3) difficulties in social roles (e.g., stress at work/school, sense of inefficiency as a worker, a student, or a stay-at-home parent). As suggested in the OQ-45 manual, a total score was computed by summing the values of all 45 items (range 0–180). Higher scores indicate higher psychological distress, with a clinical cut-off score of 63 (Lambert et al., 2004b). Given that this instrument was designed to screen a wide range of symptoms and to support referral to psychological services (if necessary), it is appropriate for use in non-clinical populations. Authors evaluating the psychometric properties of the Outcome Questionnaire found adequate stability, high internal consistency (α = 0.93), moderate to high concurrent validity and good construct validity for the overall score (Lambert et al., 1996). In this study, the coefficient for internal consistency was α = 0.96 for fathers and α = 0.94 for mothers. The French version of the questionnaire has been validated in previous studies (e.g., Flynn et al., 2002; Brosseau-Liard et al., 2020).

#### Child social-emotional maladjustment

The Strengths and Difficulties Questionnaire (SDQ; Goodman, 1997) was used to assess the child’s social-emotional maladjustment. Mothers and fathers independently screened their child’s behavior over the past 6 months, by answering 25 questions on a 3-point scale (1 = not true; 2 = somewhat true; 3 = certainly true). The SDQ is composed of five subscales, each measured by five questions relating to emotional symptoms, conduct problems, hyperactivity/inattention, peer problems, and prosocial behavior. The questionnaire allows combining the values of the first four subscales into a total behavior problems score, which was used in the current study. Given the high intercorrelation between mothers’ and fathers’ total scores (*r* = 0.66, *p* < 0.001; α = 0.80), and in line with Dubois-Comtois et al.’s (2021) study, we computed a mean score of their ratings in an effort to avoid problems associated with single informants, such as a tendency to overreport or underreport child symptoms (Alakortes et al., 2017). The SDQ has good psychometric properties, evidenced by good concurrent and discriminant validity (Goodman, 2001; Stone et al., 2010), as well as an internal consistency coefficient of 0.82 and a retest stability coefficient of 0.72 for parents’ total scores (Goodman, 2001). In this study, good internal consistency (α = 0.86) was found for parents’ mean scores. This questionnaire has been submitted to a forward/backward translation procedure and has been validated in French (for more information, see https://www.sdqinfo.org/a0.html).

#### Quality of the sibling relationship

The quality of the sibling relationship was assessed during a home visit. Siblings were asked to play Jenga together for 10 min, each taking turns trying to remove a piece of wooden block from a tower of blocks and placing it at the top of the structure, without it falling. Although the research assistant explained the purpose of the game, children could play as they pleased, given that success or compliance with the task was not taken into consideration. Parents were also asked to leave the room for the duration of the task. Video-recordings of the targeted child’s interactions with his/her siblings (regardless of the number of siblings) were coded by two independent coders using a coding system developed and adapted from the Sibling Inventory of Behavior Questionnaire (SIB; Schaefer and Edgerton, 1981). We chose this measure since it is often used by researchers (Volling and Blandon, 2003; Kolak and Volling, 2011; Yaremych and Volling, 2020) and is intended to assess a child’s interactions with his/her siblings, as opposed to assessing siblings’ overall interactions (i.e., without targeting a specific child). The coding system, previously described and published in another study (Woychuk et al., 2021), included six dimensions of the sibling relationship: (1) companionship (e.g., the child accepted his/her siblings as playmates, had fun with them during the task), (2) empathy (e.g., the child wanted siblings to succeed in the activity, comforted them when they were upset, such as when the Jenga structure collapsed), (3) teaching (e.g., the child helped his/her siblings choose which blocs to remove and where to place them on the structure), (4) rivalry (e.g., the child was competitive or jealous of his/her siblings, blamed them when the Jenga structure collapsed), (5) aggression (e.g., the child teased siblings during the task, got angry or argued with them), and (6) avoidance (e.g., the child stood at a distance, acted ashamed of his/her siblings). Each dimension was coded using a 4-point scale, ranging from 1 (very atypical of the child) to 4 (very typical of the child). Intra-class correlations performed on the original sample (*n* = 63) were greater than 0.77 for all subscales, suggesting excellent interrater reliability. In order to reduce the number of analyses performed, and in line with previous studies (e.g., Volling and Blandon, 2003), composite scores of positivity and negativity were calculated by summing the values of the three positive subscales (companionship, empathy, and teaching) and the three negative subscales (rivalry, aggression, and avoidance). However, in the event of significant results, *post hoc* analyses were performed to determine the specific contribution of each of the subscales included in the composite score.

### Statistical analyses

Descriptive statistics and bivariate correlations were calculated among variables prior to conducting the main analyses. Normality for each variable was also assessed by verifying that skewness and kurtosis values were ≤ | 2.0| (George and Mallery, 2016). Next, we explored potential control variables that are known to be related to parental and/or child social-emotional adjustment such as child gender, family annual gross income, parents’ highest level of education, and the number of siblings in the family. Sociodemographic variables that were significantly correlated with an outcome measure were included as covariates in the main analyses. Finally, we tested the moderating role of the quality of the sibling relationship in the association between parental psychological distress and child adjustment using the PROCESS macro command in SPSS (version 3.5; Hayes, 2017). We examined interaction effects at low (−1 *SD*), moderate (mean), and high (+1 *SD*) levels of sibling relationship positivity and negativity (Aiken and West, 1991). All predictors were mean centered prior to analyses. We used 95% bootstrap confidence intervals, with 5,000 bootstrap replications for each analysis.

## Results

### Control variables

Among sociodemographic variables, only child gender was significantly correlated with one dimension of the outcome variable (*r* = −0.42, *p* = 0.002). A *T*-test revealed that boys had significantly higher hyperactivity/inattention scores compared to girls, *t*(50) = 3.01, *p* = 0.005. Other studies also found gender differences in children’s prosocial behavior and behavioral problems assessed using the SDQ (Muñoz-Silva et al., 2020). Child gender was also significantly correlated with one dimension of the moderator variable (*r* = −0.28, *p* = 0.04). A *T*-test revealed that boys had significantly higher Rivalry scores compared to girls, *t*(50) = 2.07, *p* = 0.043. Accordingly, child gender was included as a covariate in the current study’s main analyses.

### Descriptive analyses

Table 1 presents bivariate correlations among variables of interest, as well as means and standard deviations for each variable. Perhaps not surprisingly when considering the low-risk status of the sample, most mothers and fathers did not report high levels of psychological distress (*M* = 42.29; *SD* = 21.06; *M* = 44.21; *SD* = 24.15, respectfully), although the scores of 11 mothers and 10 fathers were above the clinical cut-off score. The mean score for mothers’ and fathers’ ratings of child behavior problems was 8.6, with values ranging from 1 to 21.50.

**TABLE 1:** Bivariate correlations and descriptive statistics among variables of interest (*N* = 52).

Variables of interest	1	2	3	4	5	6	7	8	9	10	11
1. Maternal psychological distress (OQ-45)	–										
2. Paternal psychological distress (OQ-45)	0.43**	–									
3. Child social-emotional adjustment (SDQ)—mean of both parents’ scores	0.44**	0.33*	–								
4. Sibling positivity	0.18	−0.04	0.12	–							
5. Sibling negativity	0.04	0.01	−0.11	−0.49**	–						
6. Sibling companionship	0.21	0.08	0.18	0.72**	−0.71**	–					
7. Sibling empathy	0.09	−0.10	0.04	0.91**	−0.55**	0.67**	–				
8. Sibling teaching	0.12	−0.06	0.07	0.70**	0.05	0.07	0.46**	–			
9. Sibling rivalry	0.21	0.04	−0.09	−0.24	0.66**	−0.25	−0.32*	−0.01	–		
10. Sibling conflict	−0.02	0.06	−0.12	−0.35*	0.85**	−0.60**	−0.47**	0.18	−0.48**	–	
11. Sibling avoidance	−0.11	−0.08	−0.02	−0.44**	0.59**	−0.65**	−0.36**	−0.06	−0.07	0.31*	–
Mean	42.29	44.21	8.56	7.81	6.57	2.67	2.49	2.64	2.35	2.37	1.85
SD	21.06	24.15	4.72	1.83	1.80	0.74	0.77	0.87	0.85	0.86	0.87
Range	9–88	7–128	1–21.50	3–12	3–10.50	1–4	1–4	1–4	1–4	1–4	1–4

*p < 0.05, **p < 0.01.

Bivariate correlations among variables of interest revealed a significant association between maternal psychological distress and paternal psychological distress (*r* = 0.43, *p* = 0.001), as well as a negative significant relation between sibling positivity and sibling negativity (*r* = −0.49, *p* < 0.001). Additionally, significant associations were found between maternal reports of psychological distress and child behavior problems (*r* = 0.44, *p* = 0.001), as well as paternal reports of psychological distress and child behavior problems (*r* = 0.33, *p* = 0.017), suggesting that higher levels of parental psychological distress are related to higher levels of child social-emotional maladjustment.

### Moderation analyses

Tests of assumptions of moderation were performed and revealed that all assumptions were met. As a result, we proceeded with the main analyses. We performed four separate multiple regression analyses to examine the potential moderating effect of sibling positivity and sibling negativity in the relation between parental psychological distress and child adjustment.

#### Paternal psychological distress

**FIGURE 1:**
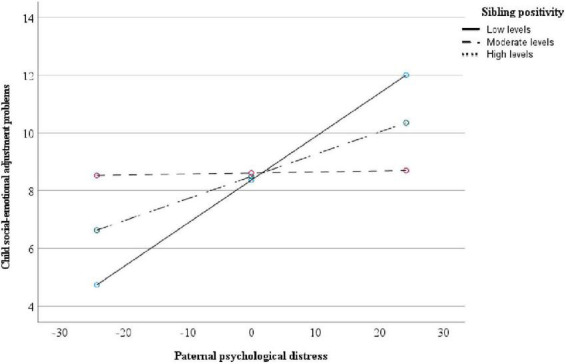
Paternal psychological distress and child adjustment problems moderated by sibling positivity (*N* = 52).

The first multiple regression model included paternal psychological distress, sibling positivity, and their interaction as predictors. Child gender was included as a covariate, whereas child social-emotional maladjustment was entered as the dependent variable. The model was significant (*R*^2^ = 0.24, *p* = 0.01), with variables accounting for 24% of the variance in child behavior problems. Whereas paternal psychological distress was a significant predictor of child maladjustment (β = 0.08, *p* = 0.005), sibling positivity was not (β = 0.07, *p* = 0.85). However, the interaction term between paternal psychological distress and sibling positivity was significant (β = −0.04, *p* = 0.04; [CI = −0.08, −0.002]). A simple slopes analysis revealed a significant association between paternal psychological distress and child maladjustment for children with low (−1 *SD*) to moderate (mean) levels of sibling positivity (β = 0.15, *p* = 0.004; β = 0.08, *p* = 0.005). In contrast, no significant association was found between paternal psychological distress and child maladjustment for children with high levels (+1 *SD*) of sibling positivity (β = 0.004, *p* = 0.92). Results are presented in Table 2 and Figure 1.

**TABLE 2:** Paternal psychological distress and child adjustment problems moderated by sibling positivity (*N* = 52).

	*b*95% CI [LL, UL]	*SE B*	*t*	*p*
Constant	12.28 [7.97, 16.59]	2.14	5.73	*p* < 0.001
Paternal psychological distress	0.077 [0.02, 0.13]	0.026	2.93	*p* = 0.005
Sibling positivity	0.066 [−0.64, 0.77]	0.35	0.19	*p* = 0.851
Paternal psychological distress × Sibling positivity	−0.04 [−0.08, −0.002]	0.019	−2.12	*p* = 0.039
Child gender (covariate)	−2.31 [−4.85, 0.224]	1.26	−1.83	*p* = 0.073

We further analyzed this interaction while controlling for the effects of maternal distress, in addition to child gender. Although the model reached statistical significance (*R*^2^ = 0.32, *p* = 0.003), the interaction was non-significant (β = −0.03, *p* = 0.07), perhaps due to insufficient statistical power.

##### *Post hoc* analyses

**FIGURE 2:**
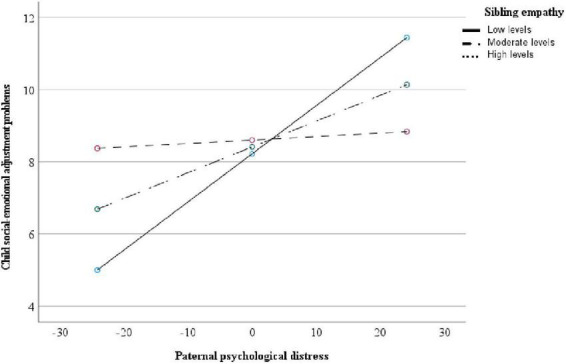
Paternal psychological distress and child adjustment problems moderated by sibling empathy (*N* = 52).

With regards to the significant interaction effect between paternal psychological distress and sibling positivity on child maladjustment, *post hoc* analyses were conducted to determine the specific contribution of each of the subscales included in the sibling positivity composite score. The model was significant for all three subscales, namely sibling companionship (*R*^2^ = 0.18, *p* = 0.05), sibling empathy (*R*^2^ = 0.24, *p* = 0.01), and sibling teaching (*R*^2^ = 0.19, *p* = 0.04). However, only the interaction effect for sibling empathy was significant (β = −0.08, *p* = 0.03). A simple slopes analysis revealed a significant association between paternal psychological distress and child maladjustment for children with low (−1 *SD*) to moderate (mean) levels of sibling empathy (β = 0.13, *p* = 0.002; β = 0.07, *p* = 0.008). Alternatively, no significant association was found between paternal psychological distress and child maladjustment for children with high levels (+1 *SD*) of sibling empathy (β = 0.01, *p* = 0.78). Results are presented in Table 3 and Figure 2.

**TABLE 3:** Paternal psychological distress and child adjustment problems moderated by sibling empathy (*N* = 52).

	*b*95% CI [LL, UL]	*SE B*	*t*	*p*
Constant	11.82 [7.55, 16.10]	2.12	5.56	*p* < 0.001
Paternal psychological distress	0.0715 [0.02, 0.12]	0.026	2.79	*p* = 0.008
Sibling empathy	0.249 [−1.34, 1.838]	0.79	0.32	*p* = 0.754
Paternal psychological distress × Sibling empathy	−0.08 [−0.152, −0.009]	0.036	−2.25	*p* = 0.029
Child gender (covariate)	−2.086 [−4.6, −0.43]	1.25	−1.67	*p* = 0.102

The second multiple regression model examined the moderating role of sibling negativity between paternal psychological distress and child social-emotional maladjustment. Child gender was included as a covariate. The model approached statistical significance (*R*^2^ = 0.17, *p* = 0.06), with a significant main effect for paternal psychological distress on child maladjustment (β = 0.063, *p* = 0.022). Conversely, the interaction effect was non-significant (β = 0.01, *p* = 0.49), and remained non-significant when controlling for the effects of maternal distress.

#### Maternal psychological distress

The third multiple regression model included the following predictors: maternal psychological distress, sibling positivity, and their interaction. Child gender was included as a covariate, whereas child social-emotional maladjustment was entered as the dependant variable. The model was significant (*R*^2^ = 0.25, *p* = 0.007). Whereas results revealed a significant main effect for maternal psychological distress on child maladjustment (β = 0.098, *p* = 0.001), the interaction effect was non-significant (β = −0.004, *p* = 0.80), and remained non-significant when controlling for the effects of paternal distress.

The fourth multiple regression model included maternal psychological distress, sibling negativity, and their interaction as predictors, child gender as a covariate, and child maladjustment as the dependent variable. The overall model was significant (*R*^2^ = 0.27, *p* = 0.004). While the interaction effect was non-significant (β = 0.0002, *p* = 0.99), results revealed a significant main effect for maternal psychological distress on child maladjustment (β = 0.10, *p* = 0.0008). The interaction effect remained non-significant when controlling for the effects of paternal distress.

## Discussion

The purpose of this study was to explore the moderating role of sibling relationship quality in the context of parental psychological distress. We extended previous findings by separately analyzing the influence of maternal and paternal psychological distress on child maladjustment and by including a multidimensional assessment of sibling interactions by independent observers. Though the use of an observational measure of the quality of the sibling relationship in the child’s natural environment limits the sample size, independent observers are likely to be more impartial than parents as they are not emotionally involved in family dynamics and are not subjected to social desirability (Woychuk et al., 2021). Considering that previous studies have mainly focused on clinical samples, we also added to previous research by recruiting families from a non-clinical population (i.e., no clinical diagnosis) with the aim of increasing the potential for generalizing the results.

The first objective of our study was to explore the association between maternal and paternal psychological distress and child social-emotional maladjustment. In line with our hypothesis, our results suggest a direct and positive association between parental psychological distress and child maladjustment for both mothers and fathers. These results are consistent with those from previous studies in which higher levels of parental psychological distress were associated with higher levels of child adjustment problems (Connell and Goodman, 2002; Dubois-Comtois et al., 2021). In contrast, the two moderating variables in this study were not significantly associated with either the independent or dependent variable. Therefore, surprisingly, child adjustment was not significantly predicted by sibling positivity nor sibling negativity. The results of a meta-analytic review (Buist et al., 2013) provide evidence that high quality sibling relationships are associated with lower levels of child internalizing and externalizing behaviors. Most studies included in this review assessed the quality of the sibling relationship by administering a questionnaire to the child or to one or both parents. In the current study, sibling interactions were assessed by independent observers in the child’s natural environment and both parents independently completed a measure on their child’s adjustment problems. Therefore, the current study is less vulnerable to a potential reporter bias compared to studies in which the same parent reported on both the sibling relationship and the child’s social adaptation. Moreover, findings from a study by Alakortes et al. (2017) support the value of considering both parents’ perspectives, as discrepancies have been found in maternal and paternal reports of child social-emotional and behavioral problems. Specifically, results suggest that mothers tend to report higher levels of child behavior problems compared to fathers (Alakortes et al., 2017), which may lead to more extreme scores in studies using single informants. Considering the small sample size of our study, further research is needed to explore the association between sibling relationship quality and child adjustment in normative samples, using multi-informant assessments.

Our second and main objective was to verify if the quality of the sibling relationship serves a protective role for children in the context of parental psychological distress. We hypothesized that the quality of the sibling relationship would play a moderating role in the association between maternal and paternal psychological distress and child maladjustment. Results provide partial support for our hypothesis. Specifically, our findings suggest that higher levels of positive sibling relationships buffer the effect of paternal psychological distress on child maladjustment, after controlling for child gender. Conversely, no moderation effect was found in relation to maternal psychological distress. Although our results are in line with those of Keeton et al. (2015) who found that quality sibling relationships moderate the effects of parental distress, the composition of their sample, involving only 19 fathers, did not allow them to perform separate analyses for fathers. In our study, separate analyses revealed that sibling relationship quality plays a moderating role only in the context of paternal psychological distress, suggesting that children of distressed mothers are at greater risk for maladjustment, even in the presence of high-quality sibling relationships.

There is evidence to suggest that the father-child relationship, as opposed to the mother-child relationship, is especially vulnerable to the influence of contextual factors (e.g., economic stress, social support, marital relationship; Doherty et al., 1998; Cabrera et al., 2000). Doherty et al. (1998) explain that cultural norms in which the mother-child relationship is viewed as being more enduring may explain why it is more resilient to the influence of environmental factors. Thus, it is possible that sibling influence occurs in the context of low-to-moderate levels of paternal psychological distress (as seen in our sample), whereas it is only apparent in the context of high levels of maternal distress. This would be consistent with findings from Keeton et al.’s (2015) study, in which parents had a clinical anxiety disorder. Additional studies are needed to test this hypothesis.

While our results indicate that sibling relationship quality moderates the association between fathers’ distress and child maladjustment, this is only the case when mothers’ levels of distress are not taken into consideration in the analyses. Perhaps siblings can only exert a positive influence on children when the child is less vulnerable to the effects of parental distress, as suggested by relatively smaller effect sizes for fathers than mothers in the current study. More research is needed to examine the influence of sibling relationship quality in intact families in which one or both parents are considered at-risk (e.g., high levels of parenting stress, hostile behaviors toward the child). This is particularly important as studies have shown that child externalizing behaviors are only associated with father-child insecurity in the context of mother-child insecurity (Bureau et al., 2020), suggesting that children are at greater risk for negative outcomes when a risk factor is present in both parents.

Considering that our measure of child positivity was composed of three subscales (i.e., companionship, empathy, and teaching), we performed *post hoc* analyses for our significant interaction. Our results revealed that high levels of sibling empathy moderate the association between paternal psychological distress and child social-emotional maladjustment, even after controlling for child gender. Conversely, no moderation effect was found for sibling companionship nor sibling teaching. Empathy is defined as one’s ability to understand and share the feelings of others (Hoffman, 2008; Eisenberg et al., 2014). In this study, sibling empathy reflected the child’s ability to support his or her siblings emotionally, such as being sympathetic or concerned about their happiness and comforting them when they were unhappy or upset. Many studies show that siblings can provide emotional support for each other in the face of adversity, such as parental conflict or parental divorce (Jacobs and Sillars, 2012; Davies et al., 2019). Therefore, it is possible that children with high-quality sibling attachment relationships are less affected by their fathers’ psychological distress, as their siblings may offer a secure presence and help them regulate their emotions (Whiteman et al., 2011). To date, several studies have been conducted with clinical samples. Our findings suggest that siblings can provide emotional support, not only for children of clinically distressed fathers, but also for children in well-functioning families. Despite the relevance of these results, additional studies are needed to clarify the proportion of variance explained by sibling empathy given its strong correlation with sibling positivity. Perhaps the observational context used in the current study encouraged greater sibling empathy, as some children can express disappointment or frustration with themselves when the Jenga tower collapses. In contrast, the simplicity of the game and its competitive nature may have resulted in less teaching and sibling companionship. Findings from this study should be replicated by varying the contexts of the observation, including settings in which sibling companionship and teaching are more prominent.

### Study implications

The results of this study have important implications for research and practice. Specifically, they emphasize the importance of including and distinguishing fathers in studies to ensure that their role in the association between parental distress and child maladjustment is not overlooked. They also stress the importance of paying attention to the social-emotional adjustment of children whose fathers are experiencing distress. Furthermore, the results of this study encourage researchers to examine family subsystems beyond the parent-child relationship, particularly the sibling subsystem. From a clinical perspective, findings support the relevance of using a systemic family approach in which a positive sibling relationship may be an interesting target for intervention to promote children’s social-emotional adaptation. Sibling interventions should focus on helping children strengthen and/or acquire empathic skills, as empathy seems to play a key role in decreasing the negative effects of parental distress. These recommendations are in line with those proposed in Kramer’s (2010) review, in which she recommends the use of a comprehensive intervention approach involving both parents and siblings. She further argues that interventions should focus on helping children develop the competencies required to engage in prosocial sibling relationships (e.g., positive communication, problem solving), rather than aiming to reduce sibling conflict (Kramer, 2010).

### Limitations and future directions

The findings of this study must be considered in light of the following limitations. First, although our study included 104 parents and their children, conducting separate analyses for mothers and fathers resulted in small sample sizes (*n* = 52). These findings must be replicated with larger samples, while still using a multi-informant approach. Larger samples would allow for more complex models (e.g., SEM) in which one could control for child gender as well as the effects of the other parent’s distress. Second, caution must be exercised in generalizing the results to families of different socioeconomic backgrounds or levels of risk as our study only involved intact families from a normative population. Nonetheless, future studies should pursue the investigation of parental distress and child maladjustment in non-clinical populations, as most studies to date have involved at-risk families. Third, although the sibling relationship was assessed by independent observers during a home visit, this relationship was only assessed on one occasion, during a play session. Future studies should investigate this relationship in different settings and at various points in time to increase the validity of the data. Finally, this study focussed exclusively on social and environmental explanatory mechanisms linking parental distress and child social and emotional functioning. Future studies should also take into consideration biological explanations, as studies show that prenatal exposure to stress is associated with child internalizing and externalizing symptoms at age 5 through child temperamental negative affect (Hentges et al., 2019).

Despite these limitations, our results make important contributions to our understanding of child social-emotional maladjustment in the context of parental psychological distress and highlight the moderating role of quality sibling relationships for children of distressed fathers. Researchers should pursue the investigation of positive sibling relationships as a potential protective factor in other family contexts (e.g., parental maltreatment).

## Data availability statement

The raw data supporting the conclusions of this article will be made available by the authors, without undue reservation.

## Ethics statement

The studies involving human participants were reviewed and approved by University of Ottawa Research Ethics Board. Written informed consent to participate in this study was provided by the participants’ legal guardian/next of kin.

## Author contributions

J-FB contributed to conception and design of the study. JT organized the database, performed the statistical analysis, and wrote the first draft of the manuscript under the supervision of J-FB. Both authors contributed to manuscript revision, read, and approved the submitted version.
